# Pitavastatin suppresses diethylnitrosamine-induced liver preneoplasms in male C57BL/KsJ-*db/db *obese mice

**DOI:** 10.1186/1471-2407-11-281

**Published:** 2011-06-28

**Authors:** Masahito Shimizu, Yoichi Yasuda, Hiroyasu Sakai, Masaya Kubota, Daishi Terakura, Atsushi Baba, Tomohiko Ohno, Takahiro Kochi, Hisashi Tsurumi, Takuji Tanaka, Hisataka Moriwaki

**Affiliations:** 1 Department of Medicine, Gifu University Graduate School of Medicine, Gifu, Japan; 2 Department of Oncologic Pathology, Kanazawa Medical University, Ishikawa, Japan

## Abstract

**Background:**

Obesity and related metabolic abnormalities, including inflammation and lipid accumulation in the liver, play a role in liver carcinogenesis. Adipocytokine imbalances, such as decreased serum adiponectin levels, are also involved in obesity-related liver tumorigenesis. In the present study, we examined the effects of pitavastatin - a drug used for the treatment of hyperlipidemia - on the development of diethylnitrosamine (DEN)-induced liver preneoplastic lesions in C57BL/KsJ-*db/db *(*db/db*) obese mice.

**Methods:**

Male *db/db *mice were administered tap water containing 40 ppm DEN for 2 weeks and were subsequently fed a diet containing 1 ppm or 10 ppm pitavastatin for 14 weeks.

**Results:**

At sacrifice, feeding with 10 ppm pitavastatin significantly inhibited the development of hepatic premalignant lesions, foci of cellular alteration, as compared to that in the untreated group by inducing apoptosis, but inhibiting cell proliferation. Pitavastatin improved liver steatosis and activated the AMPK-α protein in the liver. It also decreased free fatty acid and aminotransferases levels, while increasing adiponectin levels in the serum. The serum levels of tumor necrosis factor (TNF)-α and the expression of *TNF-α *and *interleukin-6 *mRNAs in the liver were decreased by pitavastatin treatment, suggesting attenuation of the chronic inflammation induced by excess fat deposition.

**Conclusions:**

Pitavastatin is effective in inhibiting the early phase of obesity-related liver tumorigenesis and, therefore, may be useful in the chemoprevention of liver cancer in obese individuals.

## Background

Hepatocellular carcinoma (HCC) is a serious healthcare problem worldwide because of its increasing morbidity and high mortality. Chronic inflammation of the liver and subsequent cirrhosis, which are highly correlated with hepatitis B and hepatitis C viruses infection and alcoholic liver disease, are the strongest risk factors for HCC development. Recent evidence also indicates that obesity and related metabolic abnormalities, especially diabetes mellitus and insulin resistance, raise the risk of HCC [1-4]. In obese individuals, high levels of free fatty acid (FFA) flux into the liver from excess adipose tissue. This in turn promotes hepatic steatosis and inflammation through the production of pro-inflammatory cytokines, such as tumor necrosis factor (TNF)-α and interleukin (IL)-6, and is closely associated with liver carcinogenesis [5-7]. Aberrant lipogenesis in the liver, which is closely linked to obesity and metabolic syndrome, is also a dominant event in liver carcinogenesis and human HCC progression [8]. Non-alcoholic fatty liver disease (NAFLD) is a hepatic manifestation of the metabolic syndrome and a proportion of patients with this disease can progress to non-alcoholic steatohepatitis (NASH), which involves the risk of developing cirrhosis and HCC [9]. Therefore, in addition to lifestyle modification to reduce body weight, active pharmacotherapy is considered to be necessary for the management of NASH. For instance, metformin and thiazolidinediones, both of which increase insulin sensitivity, might be useful for the treatment of patients with NASH [10].

Statins, 3-hydroxy-3-methylglutaryl coenzyme A (HMG-CoA) reductase inhibitors, are widely used for the treatment of hyperlipidemia and have been shown to reduce the risk of cardiovascular disease [11]. Statins have recently also been suggested to be possible candidates for the management of NASH/NAFLD, which frequently coexist with hyperlipidemia and cardiovascular disease [12]. A pilot study revealed that treatment with atorvastatin decreases TNF-α serum levels and improves biochemical and histological features of disease activity in NASH patients with dyslipidemia [13]. The use of atorvastatin in hyperlipidemic patients complicated with NAFLD also improves serum transaminase levels and prevents hepatic fibrosis progression [14]. In a mice model, pitavastatin, a recently developed lipophilic statin, has been shown to ameliorate severe hepatic steatosis by enhancing hepatic free acid (FA) β-oxidation activity [15].

In addition to the lipid-lowering and anti-inflammatory effects, recent studies have revealed that statins appear to have anticancer and cancer chemopreventive properties [16,17]. A large cohort study showed that statin use is associated with a reduced risk of HCC in patients with diabetes [18]. Statins inhibit cell proliferation and induce apoptosis in human HCC-derived cells [19,20]. In addition, pitavastatin prevents obesity-related colorectal carcinogenesis by correcting adipocytokine imbalance and attenuating colonic inflammation in C57BL/KsJ-*db/db *(*db/db*) mice suffering from obesity and hyperlipidemia [21]. These findings suggest the possibility that long-term use of statins may also be effective for preventing the progression of obesity-related liver tumorigenesis. Our recent study showed that diethylnitrosamine (DEN)-induced liver tumorigenesis is significantly enhanced in *db/db *mice [22]. In the present study, we examined the effects of pitavastatin on the development of DEN-induced hepatic preneoplastic lesions, foci of cellular alteration (FCA), while focusing on the improvement of liver steatosis and inflammation using a *db/db *mice model.

## Methods

### Animals and chemicals

Four-week-old male *db/db *mice were obtained from Japan SLC Inc. (Shizuoka, Japan) and were humanely maintained at the Gifu University Life Science Research Center in accordance with the Institutional Animal Care Guidelines. DEN was purchased from Sigma Chemical Co. (St. Louis, MO, USA). Pitavastatin was obtained from Kowa Pharmaceutical Co. (Tokyo, Japan).

### Experimental procedure

The animal experiment was approved by the Committee of Institutional Animal Experiments of Gifu University [22]. At 5 weeks of age, all 36 mice were administered tap water containing 40 ppm DEN for the first 2 weeks of the experiment. After DEN treatment, Groups 2 (n = 12) and 3 (n = 12) were given a basal diet (CRF-1, Oriental Yeast Co., Tokyo, Japan) containing 1 and 10 ppm pitavastatin, respectively, until the end of the experiment. Group 1 (n = 12) acted as the control and was fed only a basal diet throughout the experiment. At 21 weeks of age (after 14 weeks of pitavastatin treatment), all the mice were sacrificed to analyze the development of FCA. Since neither C57B6 nor C57BL/KsJ-+/+ mice - the genetic controls for *db/db *mice - develop FCA and liver neoplasms by DEN administration during this period [22], control experimentation using these mice was not conducted in the present study.

### Histopathology and immunohistochemical analysis for PCNA

Maximum sagittal sections of each lobe (6 sublobes) were used for histopathological examination. For all experimental groups, 4 μm-thick sections of formalin-fixed and paraffin-embedded livers were stained with hematoxylin & eosin (H&E) for histopathology. The presence of FCA, which are phenotypically altered hepatocytes showing swollen and basophilic cytoplasm and hyperchromatic nuclei, was judged according to the criteria described in a previous study [23]. The multiplicity of FCA was assessed on a per unit area (cm^2^) basis.

Immunohistochemical staining of proliferating cell nuclear antigen (PCNA), a G_1_-to-S phase marker, was performed to estimate the cell proliferative activity of FCA by using an anti-PCNA antibody (Santa Cruz Biotechnology, Santa Cruz, CA, USA) and the labeled streptavidin-biotin method (LSAB kit; DAKO, Glostrup, Denmark) [22]. On the PCNA-immunostained sections, the cells with intensively reacted nuclei were considered to be positive for PCNA, and the indices (%) were calculated in 20 FCA randomly selected from each group.

### Protein extraction and western blot analysis

Equivalent amounts of extracted mice liver proteins (20 μg/lane) were examined by western blot analysis [22]. Previously described primary antibodies for AMP-activated kinase-α (AMPK-α), phosphorylated AMPK-α (p-AMPK-α), and glyceraldehyde-3-phosphate dehydrogenase (GAPDH) were used [21], with GAPDH serving as a loading control. The primary antibody for Bad was purchased from Cell Signaling Technology (Beverly, MA, USA). The intensities of the blots were quantified with NIH Image software version 1.62.

### RNA extraction and quantitative real-time reverse transcription-PCR

Total RNA was isolated from the livers of experimental mice using the RNAqueous-4PCR kit (Ambion Applied Biosystems, Austin, TX, USA) and cDNA was amplified from 0.2 μg of total RNA using the SuperScript III First-Strand Synthesis System (Invitrogen, Carlsbad, CA, USA). Quantitative real-time reverse transcription-PCR (RT-PCR) analysis was performed using specific primers that amplify *TNF-α*, *IL-6*, *Bcl-2*, *Bad*, and *GAPDH *genes, as described previously [21,24].

### Clinical chemistry

The blood samples, which were collected at the time of sacrifice after 6 hours of fasting, were used for chemical analyses. The serum TNF-α (Shibayagi, Gunma, Japan), IL-6 (IBL, Gunma, Japan), adiponectin (Otsuka, Tokyo, Japan), and leptin (R&D Systems, Minneapolis, MN, USA) levels were determined by enzyme immunoassay according to the manufacturers' protocol. The serum levels of aspartate aminotransferase (AST), alanine aminotransferase (ALT), free fatty acid (FFA), total cholesterol, and triglyceride were measured with a standard clinical automatic analyzer (type 7180; Hitachi, Tokyo, Japan).

### Hepatic lipid analysis

Approximately 200 mg of frozen liver was homogenized, and lipids were extracted using Folch's method [25]. The triglyceride levels in the liver were measured using the triglyceride E-test kit (Wako Pure Chemical Co., Osaka, Japan) [22]. To visualize the intrahepatic lipids, Oil red O staining was utilized based on the standard procedure for frozen liver sections.

### Statistical analysis

The results are presented as means ± SD, and were analyzed using the GraphPad Instat software program version 3.05 (GraphPad Software; San Diego, CA) for Macintosh. Differences among the groups were analyzed by either one-way ANOVA or, as required, by two-way ANOVA. When the ANOVA revealed a statistically significant effect (*P *< 0.05), each experimental group was compared with the control group by using the Bonferroni multiple comparisons test. The differences were considered significant when the two-sided *P *value was < 0.05.

## Results

### General observations

As presented in Table 1, administration of pitavastatin significantly (*P *< 0.01, Group 1 vs. Groups 2 and 3) and dose dependently (*P *< 0.05, Group 2 vs. Group 3) decreased the value of body mass index (BMI). The body weight and relative weights of liver and white adipose tissue (periorchis and retroperitoneum) of the mice that received 10 ppm pitavastatin were slightly lower than those of the untreated control mice, but the differences were not significant. During the experiment, pitavastatin administration did not cause any clinical symptoms for toxicity. Histopathological examination also revealed the absence of pitavastatin toxicity in the liver, kidney, and spleen (data not shown).

**Table 1:** Body, liver, kidney and white adipose tissue weights of the experimental mice

**Group no**.	Treatment	No. of mice	Body wt (g)	**BMI**^**a**^	**Relative wt (g/100 g body wt) of**:		
					
					Liver	Kidney	**Fat**^**b**^
1	DEN alone	12	63.1 ± 7.0^c^	7.2 ± 0.6	6.4 ± 1.5	0.9 ± 0.1	9.3 ± 1.0
2	DEN + 1 ppm Pitavastatin	12	59.7 ± 3.9	6.7 ± 0.4^d^	6.0 ± 0.8	0.9 ± 0.1	9.1 ± 0.8
3	DEN + 10 ppm Pitavastatin	12	55.2 ± 9.5	6.2 ± 0.6^d,e^	5.7 ± 1.2	1.0 ± 0.2	8.7 ± 1.0

^a^Body mass index.

^b^White adipose tissue of the periorchis and retroperitoneum.

^c^Mean ± SD.

^d^Significantly different from Group 1 (*P *< 0.01).

^e^Significantly different from Group 2 (*P *< 0.05).

### *Effects of pitavastatin on DEN-induced liver preneoplastic lesions in *db/db *mice*

**Figure 1:**
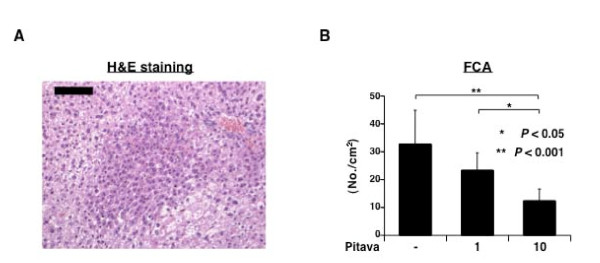
**Effects of pitavastatin on DEN-induced FCA in *db/db *mice**. (*A*) A representative photograph of FCA induced by DEN in *db/db *mice (H&E staining). Scale bar, 100 μm. (*B*) Average number of FCA in all groups (pitavastatin-untreated, 1 ppm pitavastatin-treated, and 10 ppm pitavastatin-treated groups). Each column represents the mean ± SD. * *P *< 0.05 vs. 1 ppm pitavastatin-treated group and ** *P *< 0.001 vs. the untreated group, respectively.

Liver preneoplastic lesion FCA, which possesses basophilic cytoplasm and hyperchromatic nuclei (Figure 1A), was observed in the livers of mice from all groups at the termination of the experiment. Treatment with a high dose (10 ppm) of pitavastatin significantly inhibited the development of FCA in comparison to both the untreated control mice (*P *< 0.001) and low dose (1 ppm) of pitavastatin-treated mice (*P *< 0.05). Treatment with 1 ppm pitavastatin also demonstrated a tendency to suppress the development of FCA - the inhibition rate being 29% - in comparison to the untreated control mice, but the difference did not reach a statistical significance (Figure 1B).

### *Effects of pitavastatin on the cellular levels of Bad and Bcl-2 and the proliferation activity in FCA of DEN-treated *db/db *mice*

**Figure 2:**
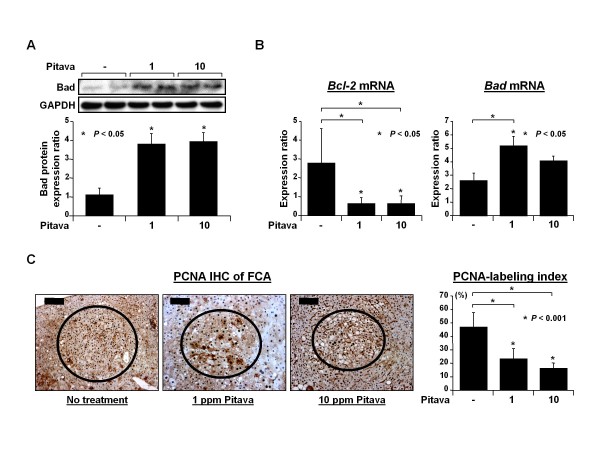
**Effects of pitavastatin on the expression levels of Bad and Bcl-2 in the liver and cell proliferation in FCA induced by DEN in *db/db *mice**. (*A*) The total proteins were extracted from the livers of experimental mice and the expression levels of the Bad protein were examined by western blot analysis (upper panel). The intensities of the blots were quantified by densitometry (lower panel). GAPDH antibody served as a loading control. Two lanes represent protein samples obtained from 2 different mice in each group. Repeat western blots yielded similar results. Values are the means ± SD. * *P *< 0.05 vs. the untreated group. (*B*) The total RNAs were isolated from the livers of experimental mice and the expression levels of *Bcl-2 *and *Bad *mRNAs were examined by quantitative real-time RT-PCR using specific primers. The expression levels of each mRNA were normalized to the level of *GAPDH *mRNA. Values represent the means ± SD. * *P *< 0.05 vs. the untreated group. (*C*) Representative pictures of the PCNA-immunohistochemical analysis of FCA (circled) developed in the livers of Groups 1, 2, and 3 (left panels). The PCNA-labeling indices of FCA developed in the livers of Groups 1 through 3 were determined by counting the PCNA-positive nuclei in FCA (right panel). Scale bars, 200 μm (no treatment) and 100 μm (1 and 10 ppm pitavastatin). * *P *< 0.001 vs. the untreated group.

We next examined the effects of pitavastatin on the induction of apoptosis in the liver and the inhibition of cell proliferation in FCA of DEN-treated *db/db *mice. Treatment with both low and high doses of pitavastatin increased the protein levels of Bad, a pro-apoptotic Bcl-2 family member, in the liver of experimental mice (Figure 2A, *P *< 0.05). The mRNA levels of this molecule were also increased by 1 ppm pitavastatin administration (Figure 2B, *P *< 0.05). On the other hand, pitavastatin treatment induced a marked decrease in the levels of an anti-apoptotic molecule Bcl-2 mRNA (Figure 2B, *P *< 0.05). In addition, as shown in Figure 2C, the mean PCNA-labeling indices for FCA in mice treated with 1 ppm (23.9 ± 7.7%) and 10 ppm (16.6 ± 4.0%) pitavastatin were significantly lower than that in the mice which received only DEN (47.7 ± 11.0%; *P *< 0.001 for each comparison). These findings indicate that pitavastatin significantly suppresses FCA, at least in part, by inducing apoptosis and by reducing cell proliferation.

### *Effects of pitavastatin on hepatic steatosis, activation of AMPK-α protein in the liver, and serum levels of FFA, total cholesterol, and triglyceride in DEN-treated *db/db *mice*

**Figure 3:**
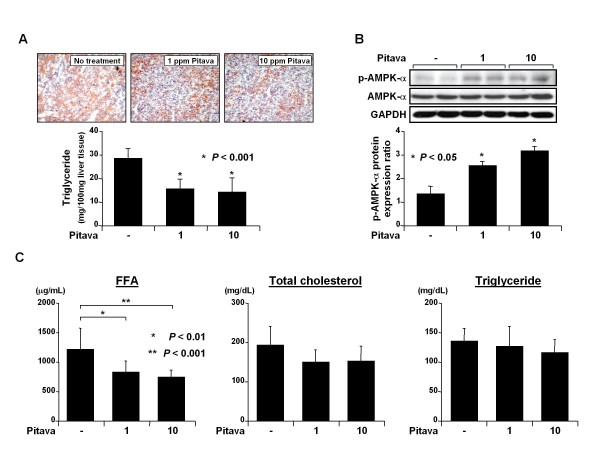
**Effects of pitavastatin on hepatic steatosis, activation of the AMPK-α protein in the liver, and serum levels of FFA, total cholesterol, and triglyceride in DEN-treated *db/db *mice**. (*A*) Frozen liver sections from experimental mice with or without pitavastatin treatment were stained with Oil red O to show steatosis (upper panels). Hepatic lipids were extracted from the frozen livers of these mice, and the triglyceride levels were measured (lower panel). Values are the means ± SD. * *P *< 0.001 vs. the untreated group. (*B*) The total proteins were extracted from the livers of experimental mice and the expression levels of the AMPK-α and p-AMPK-α proteins were examined by western blot analysis (upper panel). The intensities of the blots were quantified by densitometry (lower panel). GAPDH antibody served as a loading control. Two lanes represent protein samples obtained from 2 different mice in each group. Repeat western blots yielded similar results. Values are the means ± SD. * *P *< 0.05 vs. the untreated group. (*C*) The serum concentrations of FFA, total cholesterol, and triglyceride in all groups. Values are the means ± SD. * *P *< 0.01 and ** *P *< 0.001 vs. the untreated group, respectively.

Accumulation of lipids in the liver, which is caused by dyslipidemia, is considered to play a role in liver tumorigenesis [5,6]. Therefore, we examined whether pitavastatin improved hepatic steatosis and hyperlipidemia in the experimental mice. Examination of Oil red O stained sections revealed severe hepatic steatosis in the DEN-treated *db/db *mice; however, the mice's conditions were markedly improved by pitavastatin administration (Figure 3A, upper panels). Similar to the histological findings, the levels of intrahepatic triglyceride were also significantly reduced by administration of pitavastatin (Figure 3A, lower panel, *P *< 0.001). Western blot analysis demonstrated that pitavastatin significantly phosphorylated (*i.e.*, activated) AMPK-α - a critical kinase that monitors cellular energy status [26] - in the livers of the experimental mice (Figure 3B, *P *< 0.05). In addition, treatment with both low (*P *< 0.01) and high (*P *< 0.001) doses of pitavastatin decreased the serum levels of FFA, while the levels of total cholesterol and triglyceride were not affected by administration of this agent (Figure 3C).

### *Effects of pitavastatin on serum levels of AST, ALT, adiponectin, and leptin in DEN-treated *db/db *mice*

The serum levels of AST, ALT, adiponectin, and leptin in the experimental mice are listed in Table 2. The elevated serum AST and ALT levels, which might increase due to severe steatosis (Figure 3A), were significantly decreased by treatment with both low (*P *< 0.001) and high (*P *< 0.05) doses of pitavastatin. The serum leptin levels after pitavastatin administration demonstrated a downward trend, but the differences were not significant. However, treatment with this agent markedly increased the serum levels of adiponectin when compared to the control mice (*P *< 0.05).

**Table 2:** Serum levels of AST, ALT, adiponectin, and leptin in the experimental mice

**Group no**.	Treatment	No. of mice	**AST**^**a**^	**ALT**^**b**^	Adiponectin	Kidney
			(IU/L)	(IU/L)	(μg/mL)	(ng/dL)
1	DEN alone	12	194 ± 47^c^	291 ± 112	15.5 ± 2.4	108.1 ± 33.4
2	DEN + 1 ppm Pitavastatin	12	111 ± 28^d^	180 ± 49^d^	19.2 ± 4.5^e^	104.3 ± 33.2
3	DEN + 10 ppm Pitavastatin	12	144 ± 28^e^	227 ± 96^e^	21.2 ± 7.4^e^	93.2 ± 31.2

^a^aspartate aminotransferase.

^b^alanine aminotransferase.

^c^Mean ± SD.

^d^Significantly different from Group 1 (*P *< 0.001).

^e^Significantly different from Group 1 (*P *< 0.05).

### *Effects of pitavastatin on serum TNF-α levels and hepatic expression of *TNF-α *and *IL-6 *mRNAs in DEN-treated *db/db *mice*

**Figure 4:**
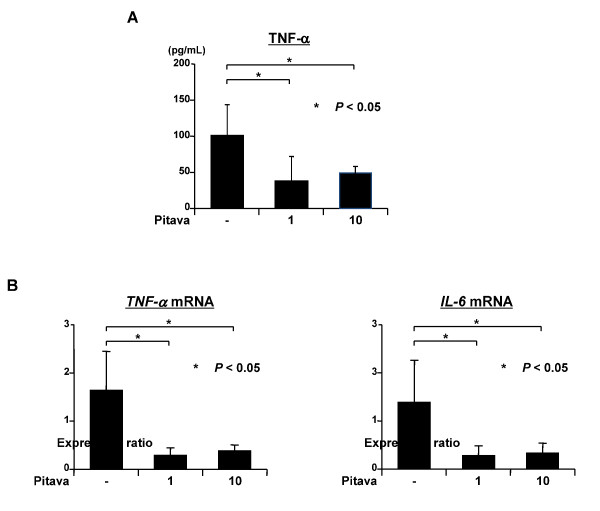
**Effects of pitavastatin on the serum levels of TNF-α and the expression levels of *TNF-α *and *IL-6 *mRNAs in the liver of DEN-treated *db/db *mice**. (*A*) The serum concentration of TNF-α was measured by enzyme immunoassay. Values represent the means ± SD. * *P *< 0.05 vs. the untreated group. (*B*) The total RNAs were isolated from the livers of experimental mice and the expression levels of *TNF-α *and *IL-6 *mRNAs were examined by quantitative real-time RT-PCR using specific primers. The expression levels of each mRNA were normalized to the level of *GAPDH *mRNA. Values represent the means ± SD. * *P *< 0.05 vs. the untreated group.

Chronic inflammation induced by excessive production of storage lipids is closely associated with obesity-related liver carcinogenesis [5-7]. Therefore, the effects of pitavastatin on the serum levels of TNF-α, a central mediator of chronic inflammatory disease, and on the expression of *TNF-α *and *IL-6 *mRNAs in the liver of DEN-treated *db/db *mice were examined. Administration of both doses of pitavastatin significantly decreased serum TNF-α levels (Figure 4A, *P *< 0.05). Further, quantitative real-time RT-PCR revealed that the expression levels of *TNF-α *and *IL-6 *mRNAs in the livers of experimental mice were also significantly decreased after pitavastatin treatment (Figure 4B, *P *< 0.05, respectively), suggesting that pitavastatin attenuated liver inflammation in obese *db/db *mice.

## Discussion and Conclusions

Statins lessen hyperlipidemia by competitively inhibiting HMG-CoA reductase, and thus, they are effective in preventing cardiovascular disease [11]. On the other hand, many studies have shown the anticancer and cancer chemopreventive effects of statins, such as the inhibition of cell proliferation, promotion of apoptosis, and inhibition of inflammation, angiogenesis, and metastasis [16,17,19,20]. The anticancer effects of statins also involve the inhibition of geranylgeranylation, primary of the Rho proteins [16,17]. These findings suggest the possibility of statins playing a role of cancer chemopreventive agents for certain malignancies.

The results of the present study clearly indicated that pitavastatin, which is widely used for the treatment of patients with hyperlipedemia, effectively prevents the development of DEN-induced liver preneoplastic lesions in obese *db/db *mice (Figure 1B). This is the first report that shows the preventive effect of statin analog on the development of obesity-related liver tumorigenesis. The unfavorable effects of obesity and related metabolic abnormalities are serious global healthcare problem. Among them, the promotion of HCC by obesity [1-4] is one of the critical issues that need to be addressed in the management of this malignancy. Therefore, our present finding seems to be clinically significant when considering the prevention of HCC in obese people, who are at an increased risk of developing HCC.

The suppressive effect of pitavastatin on the development of obesity-related liver tumorigenesis was most likely associated with the induction of apoptosis in the liver (Figures. 2A and 2B) and the inhibition of proliferation in FCA (Figure 2C). This inhibition was also associated with the improvement of hepatic steatosis (Figure 3A) and the attenuation of inflammation (Figure 4) because excess accumulation of lipids in the liver accelerates hepatic tumorigenesis by inducing a chronic inflammatory reaction [5-7]. Pitavastatin mainly ameliorates hepatic steatosis by decreasing serum FFA levels (Figure 3C) since the high influx of FFA into the liver plays a major role in hepatic fat accumulation [5,6]. In addition, activation of AMPK-α by pitavastatin in the liver (Figure 3B), which increases FA oxidation, decreases FA synthesis, and improves hyperlipidemia [26], also contributes to the inhibition of lipid deposition in the liver. Further, these findings are significant when considering the prevention of obesity-related carcinogenesis because AMPK is regarded as a metabolic tumor suppressor and a promising target for cancer prevention and therapy [27]. AMPK activity is associated with the inhibition of lipogenesis, which has a pathogenic and prognostic significance for HCC [8], induction of apoptosis, and suppression of cell growth in human HCC-derived cells [28]. Pitavastatin has also been shown to inhibit obesity-related colorectal carcinogenesis through the activation of AMPK-α in the colonic mucosa [21].

In the present study, lipid-lowering effects of pitavastatin were positive on serum FFA but not significant on total cholesterol and triglyceride in DEN-treated *db/db *mice (Figure 3C). These findings are consistent with the results of a recent study indicating more high doses of pitavastatin (20 and 40 ppm) did not significantly decrease the serum levels of total cholesterol and triglyceride in Min mice, which show a hyperlipidemic state [29]. On the contrary, Egawa *et al*. [15] demonstrated that pitavastatin administration resulted in a significant reduction in the levels of plasma triglyceride and total cholesterol in aromatase-deficient mice. Treatment with both 1 and 10 ppm pitavastatin for 8 weeks also reduced serum levels of total cholesterol, but not triglyceride, in azoxymethane-treated *db/db *mice [21]. These reports [15,21,29], together with the results of the present study, suggest that effects of pitavastatin on plasma lipids might depend on the animal strain and experimental procedure. In addition, it has been shown that pitavastatin potently inhibits *de novo *cholesterol synthesis without affecting serum lipid levels [30,31]. In rodents, cholesterol synthesis enzymes were remarkably induced by feedback regulation [32], suggesting that the effects of pitavastatin on reduction of plasma lipid and inhibition of HMG-CoA reductase activity might be masked by such feedback regulation.

Increases in TNF-α and IL-6 levels, which are accompanied by lipid accumulation in the liver, are involved in obesity-related liver carcinogenesis [5-7]. Therefore, reduction of serum TNF-α levels (Figure 4A) and inhibition of the expression of *TNF-α *and *IL-6 *mRNAs in the liver (Figure 4B) by pitavastatin are important in preventing obesity-related liver tumorigenesis. These findings are consistent with previous reports that pitavastatin significantly suppresses inflammation- and obesity-related mouse colon carcinogenesis by attenuating chronic inflammation [21,33]. The effects of pitavastatin on decreasing the levels of TNF-α might be largely dependent on the reduction of BMI (Table 1) and serum FFA levels (Figure 3C). These phenomena may also be associated with the improvement of adipocytokine imbalance (Table 2) because TNF-α has been shown to decrease the levels of adiponectin, which is secreted by the adipose tissue, while increasing the levels of leptin in the adipocytes [34,35]. Moreover, up-regulation of serum adiponectin levels (Table 2) also plays a role in attenuating inflammation because this adipocytokine possesses the ability to down-regulate the production of TNF-α and IL-6 [36]. Adiponectin alleviates hepatic steatosis and ALT abnormalities in alcohol-induced fatty liver mice model and in *ob/ob *mice, a NAFLD mice model, by enhancing FA oxidation, while decreasing FA synthesis and TNF-α production in the liver [37]. Hypoadiponectinemia enhances the progression of steatosis and hepatic tumor formation in a mice model of NASH [38]. In addition, adiponectin inhibits cell proliferation and induces apoptosis in human HCC-derived cells by inducing AMPK activation [39]. Therefore, the elevation of adiponectin and activation of AMPK might be effective for the prevention of obesity-related tumorigenesis.

Hepatotoxicity is one of the critical concerns in treatment with statins. In the present study, however, pitavastatin did not cause significant toxicity in the liver as determined by histological examination. The serum aminotransferase (ALT and AST) levels were also decreased by treatment with this agent (Table 2). The safety of statins for patients with liver dysfunction has also been reported in several clinical trials [40]. In addition, patients with chronic liver disease, including NAFLD/NASH and HCV infection, may benefit from statins because cardiovascular risk is likely to be high in these diseases [12,41]. Therefore, statin use might be a promising therapy for NASH patients who have an increased risk of HCC [9], although periodic monitoring of serum aminotransferase levels should be conducted. The result of a recent epidemiological study revealing a significant relationship between the risk reduction of HCC and statin use among diabetic patients [18] may also encourage statin therapy for patients with chronic liver disease, especially NASH patients, who frequently have hyperlipidemia as well as insulin resistance.

Finally, it should be noted that the results of recent studies indicating that supplementation with branched-chain amino acids and acyclic retinoid, both of which exert chemopreventive effects on the development of HCC in clinical trials [3,42], suppresses DEN-induced liver tumorigenesis in *db/db *mice by improving hepatic steatosis and attenuating chronic inflammation [22,43]. In summary, the results of the present study, together with those of the cited reports [22,43], suggest that the prevention of liver carcinogenesis by targeting hepatic steatosis, chronic inflammation, and adipocytokine imbalance, through either pharmaceutical or nutritional intervention, might be a promising strategy for obese individuals who are at an increased risk of developing HCC. Pitavastatin appears to be a potentially effective candidate for this purpose since it can improve liver steatosis and attenuate inflammation, at least in part, through the activation of AMPK-α and up-regulation of adiponectin.

## List of abbreviations used

ALT: alanine aminotransferase; AMPK: AMP-activated kinase; ANOVA: analysis of variance; AST: aspartate aminotransferase; BMI: body mass index; DEN: diethylnitrosamine; FA: fatty acid; FCA: foci of cellular alteration; FFA: free fatty acid; GAPDH: glyceraldehyde-3-phosphate dehydrogenase; H&E: hematoxylin & eosin; HCC: hepatocellular carcinoma; HMG-CoA: 3-hydroxy-3-methylglutaryl coenzyme A; IL: interleukin; PCNA: proliferating cell nuclear antigen; RT-PCR: reverse transcription-PCR; TNF-α: tumor necrosis factor-α.

## Competing interests

The authors declare that they have no competing interests.

## Authors' contributions

MS, YY, and TT conceived of the study, participated in its design, and drafted the manuscript. MS, YY, HS, MK, DT, AB, and TO performed *in vivo *experiment. TK performed statistical analysis. HT and HM helped to draft the manuscript. All authors read and approved the final manuscript.

## Pre-publication history

The pre-publication history for this paper can be accessed here:

http://www.biomedcentral.com/1471-2407/11/281/prepub
